# Umami the Fifth Basic Taste: History of Studies on Receptor Mechanisms and Role as a Food Flavor

**DOI:** 10.1155/2015/189402

**Published:** 2015-07-12

**Authors:** Kenzo Kurihara

**Affiliations:** Aomori University, Aomori 030-0943, Japan

## Abstract

Three umami substances (glutamate, 5′-inosinate, and 5′-guanylate) were found by Japanese scientists, but umami has not been recognized in Europe and America for a long time. In the late 1900s, umami was internationally recognized as the fifth basic taste based on psychophysical, electrophysiological, and biochemical studies. Three umami receptors (T1R1 + T1R3, mGluR4, and mGluR1) were identified. There is a synergism between glutamate and the 5′-nucleotides. Among the above receptors, only T1R1 + T1R3 receptor exhibits the synergism. In rats, the response to a mixture of glutamate and 5′-inosinate is about 1.7 times larger than that to glutamate alone. In human, the response to the mixture is about 8 times larger than that to glutamate alone. Since glutamate and 5′-inosinate are contained in various foods, we taste umami induced by the synergism in daily eating. Hence umami taste induced by the synergism is a main umami taste in human.

## 1. Introduction

In 1908, the active principle of seaweed* kombu *was identified as glutamate by Ikeda [1]. Taste of glutamate is uniquely different from classical 4 basic tastes and he termed it umami [1]. 5′-Inosinate from dried bonito [2] and 5′-guanylate from dried* shiitake *mushroom [3] were also found to have umami taste. Later umami substances have been found universally in various foods. In human, there is a large synergism between glutamate and 5′-inosinate or 5′-guanylate. However, since umami substances alone have a rather weak umami taste, umami taste is not accepted in Europe and America for a long time. Umami substances have been considered to be “flavor enhancer.”

Before the First International Symposium on Umami was held in Hawaii, there were a number of problems in umami. There was no systematic psychophysical data on umami. In electrophysiological studies, monosodium glutamate (MSG) is usually used as an umami stimulus since glutamic acid itself has no umami taste. Single taste fibers which respond to MSG always respond to NaCl and hence there is no evidence indicating that there are single fibers responding only to umami stimuli. The synergism between glutamate and 5′-nucleotides was seen in rodents, but magnitude of the synergism was extremely lower than that in human.

After the International Symposium, the psychophysical and electrophysiological studies showed that umami is independent of the four classical basic tastes. In addition, dog showed a large synergism comparable to that in human. Furthermore, mGluR1, mGluR4, and T1R1 + T1R3 were identified to be receptors for umami taste. Based on these studies, umami was internationally recognized as the fifth basic taste.

About 20 g/day of glutamate which comes from free glutamate in foods and digestion of proteins in foods is adsorbed at small intestine. Most glutamate adsorbed is used as major oxidative fuel for the gut, metabolized into other nonessential amino acids and production of glutathione. That is, dietary glutamate does not go to tissues such as brain and muscle.

## 2. Discovery of Umami Substances

**Figure 1:**
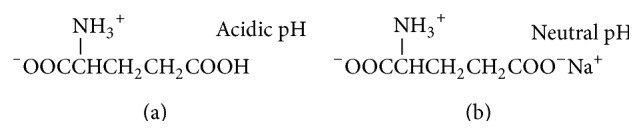
Structure of glutamic acid (a) and monosodium glutamate (b).

The seaweed* kombu* has been used as a material to make* dashi* (soup stock) in Japan for a long time. In 1908, Ikeda who was a professor of physical chemistry in University of Tokyo began to identify the active principle in* kombu* and identified the principle in the same year [1]. He used 12 kg of dried* kombu* and extracted the principle with water [1]. At acidic condition, he obtained crystals of glutamic acid (Figure 1), but glutamic acid itself has sour taste. Glutamic acid has two carboxyl residues as shown in Figure 1. pKa of *γ*-carboxyl residue is 4.25 and then this residue is COO^−^ at neutral pH. Glutamic acid dissolved in water was neutralized with NaOH and 30 g of crystals of monosodium glutamate (MSG) was obtained. MSG has unique taste different from classical 4 basic tastes (sweet, bitter, sour, and salty tastes). He termed taste of MSG umami. Potassium glutamate and calcium glutamate also have umami taste and then umami taste is due to glutamate anion.

Dried bonito has been used to make* dashi *in Japan for a long time. In 1913, Kodama who was the best pupil of Ikeda found that the active principle of dried bonito is 5′-inosinate (salt of 5′-inosinic acid) [2]. 5′-Inosinic acid is a nucleotide and has phosphate residue. At neutral pH, 5′-inosinate is an anion. Similar to glutamate, anion form of 5′-inosinic acid has umami taste.

In 1957, Kuninaka found that 5′-guanylate has umami taste [3]. 5′-Guanylate is also a nucleotide which has phosphate residue. At neutral pH, 5′-guanylate is an anion. Later, it was found that 5′-guanylate is an umami component in* shiitake* mushroom.

## 3. Production and Decomposition of Umami Substances

### 3.1. Glutamate

Free glutamate exists in various foodstuffs as shown in Table 1 [10]. Proteins are composed of 20 different amino acids. Most proteins contain glutamate in high content. For example, glutamate contents of casein in milk, gluten in wheat, glycinin in soybean, and myosin in muscle are 21–35%. Although free glutamate has umami taste, glutamate in proteins has no taste. Proteolysis during fermentation produces free glutamate in high content.

Free glutamate is not easily broken by heating and then is rather stable.

### 3.2. 5′-Inosinate

5′-Inosinate is produced by decomposition of ATP (adenosine triphosphate). ATP is decomposed into AMP which is further decomposed into 5′-inosinate. Production of 5′-inosinate begins when animal is dead and slowly proceeds. In the case of yellowtail, decomposition of ATP and production of 5′-inosinate begin at the time of killing and the concentration of 5′-inosinate reaches maximum level about 10 hours after killing. This means that the fish is not delicious just after killing but becomes delicious about 10 hours after killing:

(1) $$\begin{array}{c} \text{ATP}⟶\text{AMP}⟶5^{\prime }\text{-Inosinate} \end{array}$$

5′-Inosinate is not easily broken by heating.

### 3.3. 5′-Guanylate

5′-Guanylate is produced by decomposition of ribonucleic acid. In living cells, ribonucleic acid does not contact with ribonuclease and then the decomposition does not occur. When cells are dead, cells are broken and ribonuclease contacts with ribonucleic acid. Then 5′-guanylate is produced. Optimum temperature of the enzyme is 60~70°C. 5′-Guanylate is decomposed into guanosine by nucleotidase. The optimum temperature of this enzyme is 45~50°C:

(2) Ribonucleic acid→ribonucleaseribonuclease5′-Guanylate→nucleotidasenucleotidaseGuanosine

5′-Guanylate as an umami substance was first found in dried* shiitake *mushroom. Content of 5′-guanylate in a raw mushroom is rather low but is very high in dried mushroom. In the process of drying, cells of mushroom are broken and 5′-guanylate is produced by decomposition of ribonucleic acid by ribonuclease. Before cooking, the dried mushroom is soaked in water. The water should be cold because 5′-guanylate is decomposed into guanosine by nucleotidase at room temperature. In cooking, temperature of the water containing 5′-guanylate from the mushroom should be quickly increased to 60~70°C to produce 5′-guanylate further.

## 4. Content of Umami Substances in Various Foodstuffs

As described above, there are 3 umami substances, glutamate, 5′-inosinate, and 5′-guanylate. Contents of these substances in various foodstuffs have been measured. The contents vary with state of preservation and aging and with measurement method. The data shown in Table 1 are most reliable ones at present [10].

Glutamate is contained universally in both plant and animal foodstuffs.* Kombu* and seaweed* nori *contain glutamate in very high content. Among vegetables, tomato and tamarillo, which is relative to tomato, contain glutamate in most high contents. Animal foodstuffs also contain glutamate, but the contents are relatively lower than those in plant foodstuffs. Fermented foods contain high content of glutamate brought about by hydrolysis of proteins during fermentation. 5′-Inosinate is contained only in animal foodstuffs. Particularly, dried foodstuffs such as dried sardine and bonito contain 5′-inosinate in high content. 5′-Guanylate is contained mainly in mushrooms.

## 5. Synergism between Glutamate and the Nucleotides

Synergism between glutamate and 5′-nucleotides (5′-inosinate and 5′-guanylate) was found by Kuninaka [3]. Kuninaka first tasted 5′-inosinate and felt that umami taste of 5′-inosinate is rather weak and then tasted glutamate. He felt that umami taste of glutamate is much stronger than that of 5′-inosinate. To confirm this fact, he tasted 5′-inosinate again without rinsing his mouth. Surprisingly, he felt a very strong umami taste. This was because 5′-inosinate was mixed with glutamate remaining on the tongue. Such synergism also occurs between glutamate and 5′-guanylate.

**Figure 2:**
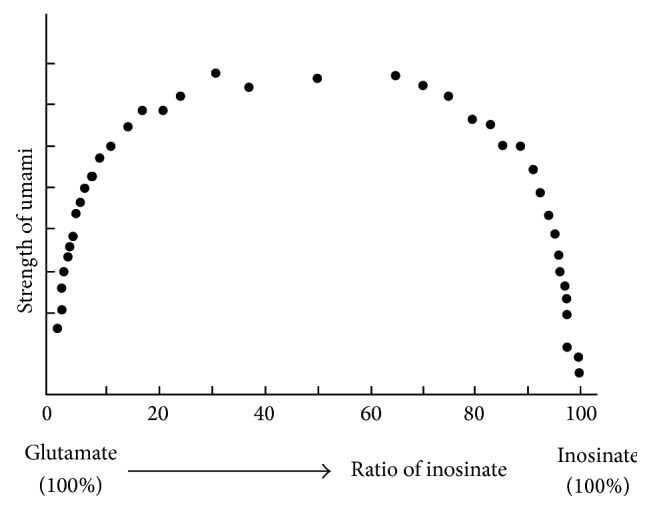
Effects of addition of  5′-inosinate to glutamate on strength of umami [4].

Figure 2 shows strength of umami taste against the ratio of glutamate and 5′-inosinate [4]. Strength of umami taste of glutamate alone is rather weak (left end of the graph). Increase of ratio of 5′-inosinate brings about a very strong umami taste. Strength of umami taste of 5′-inosinate alone (right end of the graph) is rather weak. Thus umami taste induced by the synergism is extremely strong and the synergism is essentially important.

In rats, the synergism occurs between 5′-inosinate and various amino acids including glutamate. The response of chorda tympani nerve to glutamate and 5′-inosinate is enhanced by about 1.7 times [12]. Thus the extent of the synergism in rats is much smaller than that in human.

**Figure 3:**
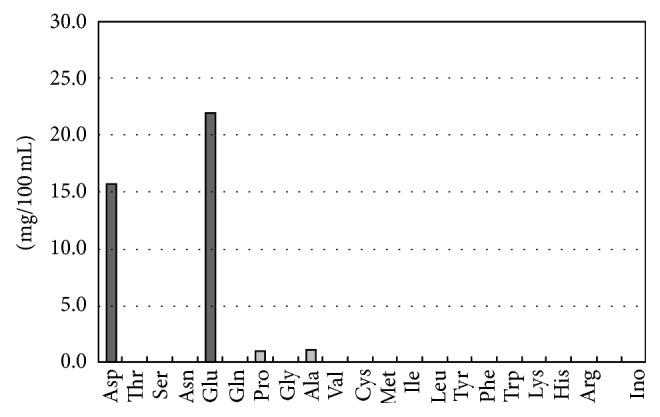
Amino acid composition of* kombu dashi *[5].

Empirically, the synergism has been used in the cooking before Kuninaka's finding. To make the best* dashi *(soup stock) in Japan, the seaweed* kombu* containing glutamate and dried bonito containing 5′-inosinate are used together. First* kombu *is soaked in water at 60°C for one hour.  Figure 3 shows amino acid composition of* kombu dashi *obtained. Surprisingly, the* kombu dashi* contains only glutamate and aspartate [5]. Aspartate is also an umami substance, although its umami taste is much weaker than that of glutamate. Thus the* kombu dashi* is a pure umami solution. Mother milk contained high content of glutamate [13]. The concentration of glutamate in mother milk is of similar level to that of the* kombu dashi*.

Secondly flaks of dried bonito are added to the* kombu dashi *and the flaks are eliminated soon after boiled. The* dashi *obtained contains 5′-inosinate and histidine in addition to glutamate and aspartate from* kombu*. This* dashi* has strong umami taste due to synergism between glutamate and 5′-inosinate. It is noted that mother milk contains 5′-inosinate as well as glutamate and hence synergism between glutamate and 5′-inosinate works in the milk.

*Kombu* and dried bonito are not easily available in countries other than Japan. In this case, tomato or dried one instead of* kombu* and the mushrooms containing 5′-guanylate such as dried porcini and morel instead of dried bonito can be used.

The synergism has been used in cooking in all over the world. In China, chicken which contains 5′-inosinate and vegetables containing glutamate such as spring onion and ginger are cooked together. In Europe and America, beef which contains 5′-inosinate is used together with vegetables containing glutamate such as onion, carrot, celery, or tomato.

Glutamate and 5′-inosinate are commercially available and hence a mixture of glutamate and 5′-inosinate produces a strong umami taste, which is similar to the umami taste brought about by combination of natural foods. Safety of glutamate was confirmed as described later.

## 6. The Umami, Amino Acid, and Sodium Chloride Interplay

Fuke and Konosu [11] determined essential components of snow crab meat taste by the omission test. First chemical compositions of the boiled crab meat were analyzed. A mixture of pure chemicals of the crab meat components has a taste similar to crab meat taste. Omission of some components still elicits crab meat taste, but that of some components does not elicit crab meat taste anymore. Thus essential components of crab meat taste were determined.

As shown in Table 2 [11], three amino acids (glycine, alanine, and arginine), two umami substances (glutamate and 5′-inosinate), and two salts (NaCl and K_2_HPO_4_) are essential components for the crab meat taste. According to our experience, K_2_HPO_4_ does not so contribute to crab meat taste.

Essential components of many other foods are also amino acids, umami substances, and salts. For example, a scallop is a sweet shellfish because it contains sweet amino acid, glycine in very high content (1,925 mg/100 g) together with alanine and arginine. Sea urchin eggs have unique taste which is due to methionine. Thus species and content of amino acids contribute to characteristic taste of foods.

Elimination of the umami substances from the essential components of the crab meat taste leads to loss of delicious taste of the crab meat. Umami substances give deliciousness to foods.

Elimination of NaCl from the components of crab meat taste brings about a very weak taste. That is, NaCl has an essential component to enhance tastes of other components.

**Figure 4:**
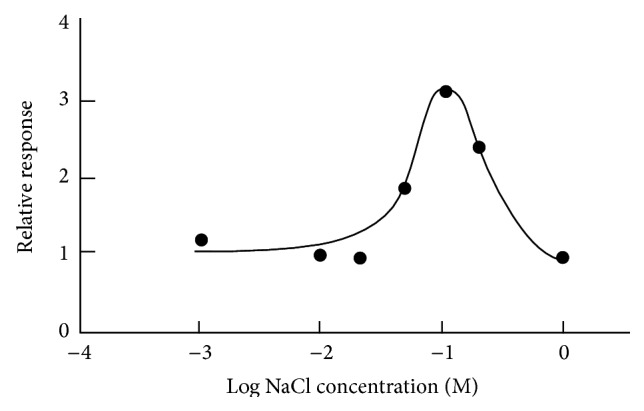
Canine taste nerve response to 100 mM glycine as a function of NaCl concentration [6].

In order to clarify the enhancing effect of NaCl, the effect of NaCl on sweet taste of glycine was examined psychophysically [14]. The results show that sweet taste of glycine is greatly enhanced by the presence of NaCl. To confirm the enhancement of NaCl more quantitatively, the recording of canine chorda tympani nerve (taste nerve) was carried out [6].  Figure 4 shows that the response to glycine is greatly enhanced by adding of NaCl. Maximum enhancing effect is seen at 100 mM (0.6%) NaCl. One hundred mM NaCl itself has only weak saltiness. Further increase of NaCl concentration decreases the enhancement. The enhancement by NaCl was also seen with other amino acids.

**Figure 5:**
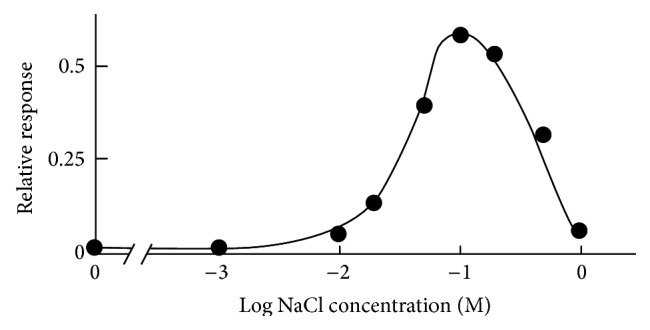
Canine taste nerve response to 100 mM glutamate as a function of NaCl concentration [7].

The responses to umami substances such as glutamate, 5′-inosinate, and 5′-guanylate were also enhanced by NaCl [7].  Figure 5 shows the enhancing effect of NaCl on the response to glutamate. Maximum enhancing effect is also seen at 100 mM NaCl. Thus NaCl of rather low concentration is essentially important for tastes of foods.

First role of the umami substances is to give umami taste itself. As mentioned in Section 4,* kombu dashi *is a pure umami solution.* Komb dashi *and the* dashi *made from* kombu* and dried bonito have a pure umami taste. Second role of the umami substances is to give deliciousness to foods. For example, elimination of the umami substances from essential components for crab meat taste lost deliciousness of crab meat taste.

Rolls [15] showed that odor together with glutamate brings about pleasantness in primates. When glutamate is given in combination with a consonant, savory odor (vegetable), the resulting flavor, formed by a convergence of the taste and olfactory pathways in the orbitofrontal cortex, can be much more pleasant.

There are* kokumi* taste substances which themselves have no taste but an ability to enhance umami, sweet, and salty tastes [16]. Various extracellular calcium-sensing receptor agonists *γ*-glutamyl peptides such as *γ*-Glu-Cys-Gly and *γ*-Glu-Val-Gly are* kokumi* taste substances. Since these* kokumi* taste substances are contained in foods, the substances contribute to taste of foods.

## 7. Umami Was Recognized as the Fifth Basic Taste

All umami substances were found by Japanese scientists and hence umami taste has been well accepted by the Japanese. However in Europe and America, umami taste has not been accepted for a long time. Glutamate itself has been considered to have no taste and the ability to enhance food flavors. Then glutamate has been called “flavor enhancer.” In these times, no original paper on umami taste has been accepted in any journal published in America and Europe.

In 1982, Japanese scientists who have studied on umami established “Umami Research Organization.” This organization held international umami symposiums in Hawaii [17], Sicily [18], Bergamo [19], and Tokyo [20]. In addition, the umami section was provided in International Symposium on Olfaction and Taste (ISOT) from 11th ISOT (1993) held in Sapporo.

In Hawaii, Yamaguchi [21] reported psychophysical data on umami taste. She examined similarities among 21 taste stimuli and showed that the four basic tastes (sweet, sour, salty, and bitter) are located at the four vertices of a three-dimensional tetrahedron and umami is located clearly apart from any vertices of the tetrahedron. This implies that umami taste is different from the four basic tastes. It was confirmed that umami has no ability to enhance any basic tastes.

Since glutamate has umami taste, monosodium glutamate (MSG) is usually used as an umami stimulus in electrophysiological studies. Application of MSG to tongue elicits impulses in taste nerve fibers. Single taste nerve fibers which respond to MSG always respond to NaCl and hence the response to MSG has been considered to be due to Na^+^ contained in MSG. Then it has been considered that there is no single fiber specific to umami substances.

Ninomiya and Funakoshi [22] showed that there are single fibers in mice glossopharyngeal nerves which respond to MSG but respond only poorly to NaCl. Baylis and Rolls [23] measured responses of single nerve fibers in the macaque taste cortex and found single fibers which responded best to glutamate.

**Figure 6:**
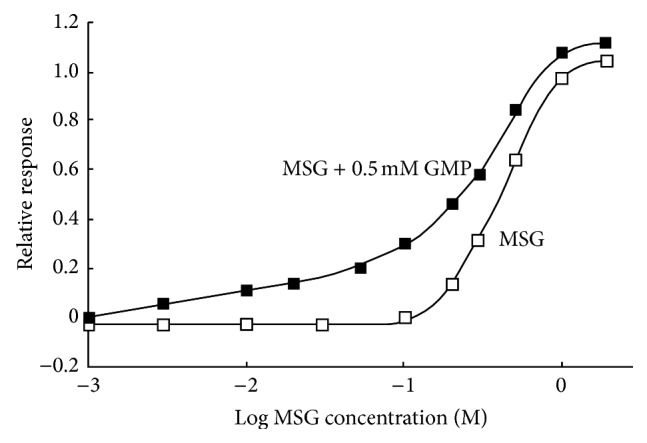
Canine taste nerve response to monosodium glutamate (MSG) in the absence and presence of 0.5 mM 5′-guanylate (GMP) [8].

Synergism between glutamate and the 5′-nucleotides in human is extremely large as shown in Figure 2. However, the synergism of rat taste nerves [12] is much smaller than that in human. We found that canine chorda tympani nerves showed a large synergism between glutamate and 5′-guanylate [8].  Figure 6 shows the responses as a function of MSG concentration in the absence and presence of 0.5 mM 5′-guanylate (GMP). GMP (0.5 mM) alone and a low concentration of MSG alone do not elicit the response. But an increase of MSG concentration induces a large response even at concentration where MSG alone does not elicit the response. This large synergism is similar to that in human.

**Figure 7:**
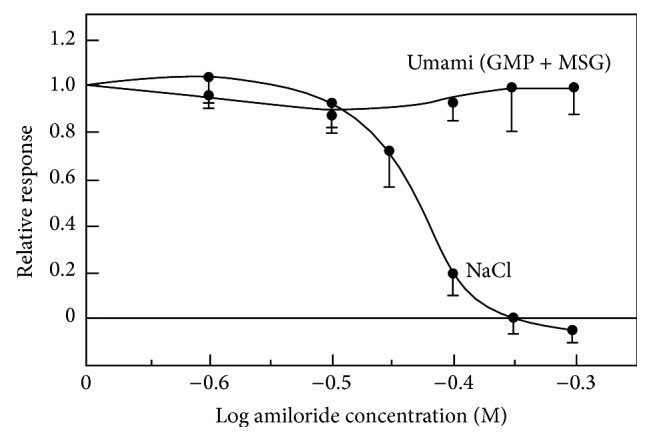
Canine taste nerve response to a mixture of 100 mM monosodium glutamate (MSG) and 0.5 mM 5′-guanylate (GMP) and 100 mM NaCl as a function of amiloride concentration [9].

In order to clarify whether the responses to umami substances are due to Na^+^ or not, amiloride which is an inhibitor for NaCl response was added to a mixture of MSG and 5′-GMP [9]. The large response brought about by the synergism was not affected by amiloride (Figure 7). This implies that the responses to the umami substances are pure umami responses.

In 1997, umami section was provided in 12th ISOT held in San Diego. In this section, many interesting data were presented. The important topic was that a candidate for umami receptor was proposed by Chaudhari and Roper, which will be described in detail later.

Since the first umami symposium, data indicating that umami taste is a basic taste have been accumulated. The conditions of a basic taste are as follows. (1) A basic taste should not be produced by any combination of other basic tastes. (2) That a basic taste is independent of other basic tastes should be proved by psychophysical and electrophysiological studies. (3) Specific receptor for a basic taste should exist. (4) A basic taste should be found universally in many foods.

Umami taste is not produced by combination of any four basic tastes. It was shown that umami taste is independent of the four basic tastes by psychophysical and electrophysiological studies. The receptor specific for umami was identified (see later). Umami substances are contained universally in many foods. Based on these facts, umami taste was recognized as the fifth basic taste.

The above results in the ISOT were announced by newspapers in all over the world and then umami became popular to ordinary people. Now the word of umami is appearing in many international dictionaries.

The basic tastes have each characteristic physiological role. Typical sweet substances are sugars, which supply energy. Hence sweet taste is a signal of energy. Poisonous substances have bitter taste in general and hence bitter taste is a signal of poison. Putrid matter has sour taste. In addition, nonripping fruits have sour taste. The seeds of mature fruits are spread through droppings of animals. Seeds of nonripping fruits cannot be germinated. To prevent nonripping fruits from eating by animals, the fruits seem to have sour taste. For animals, sour taste is a signal to protect eating putrid foods and nonripping fruits. Salts are essential elements for health and salty taste is a signal of minerals. Glutamate which is a main umami substance is most abundantly contained in proteins. Glutamate is a precursor of a protein and a component of protein hydrolysate. Then umami taste is a signal of protein.

On the contrary from the classical basic tastes, umami is not profound taste. Even high concentration of umami substances does not bring a strong taste. Umami harmonizes other tastes in foods and brings about mildness and deliciousness.

## 8. Receptors of Umami

### 8.1. mGlu4

Glutamate is a neurotransmitter in brain. There are many different types of glutamate receptors including inotropic and metabotropic receptors. Under an idea that glutamate receptors in brain may be candidates for umami receptors in taste buds, glutamate receptors in brain were looked for in rat lingual tissue [24]. A number of inotropic receptors were expressed in the lingual tissue, but no receptors were preferentially localized to taste buds. On the other hand, mGluR4 which is a member of metabotropic receptors was expressed in taste buds.

mGluR4 is a class C GPRs (G-protein coupled receptors) and has a long extracellular N terminus. mGluR4 expressed in taste buds was an unusual variant of mGluR4 [24]. That is, taste-mGluR4 expressed in taste buds lacks −50% of the receptor's extracellular N terminus. Thus taste-mGluR4 is truncated version of the mGlR4 in brain.

The concentration of glutamate to activate taste-mGluR4 is approximately two orders of magnitude less sensitive to glutamate than mGluR4 in brain whose concentration is micromolar range. The concentration of glutamate to activate umami taste is 1–3 mM in rodents and the concentration of glutamate to activate taste-mGluR4 is similar to concentration to activate umami taste. On the other hand, the synergism between glutamate and the 5′-nucleotides is not seen in taste-mGluR4.

Later mGluR1 which is a metabotropic glutamate receptor was also found in taste buds [25].

### 8.2. T1r1 + T1r3

Identification of olfactory receptors affected studies on taste receptors. Buck and Axel [26] looked for GPRs from the olfactory epithelium since cyclic AMP was established to be a second messenger in olfactory system. Similarly, GPRs from the tong epithelium were looked for. First T2Rs were identified to be receptors for bitter stimuli [27] and a heterodimeric complex of T1R2 + T1R3 was identified to be a receptor for sweet stimuli by a number of groups (e.g., [28]).

**Figure 8:**
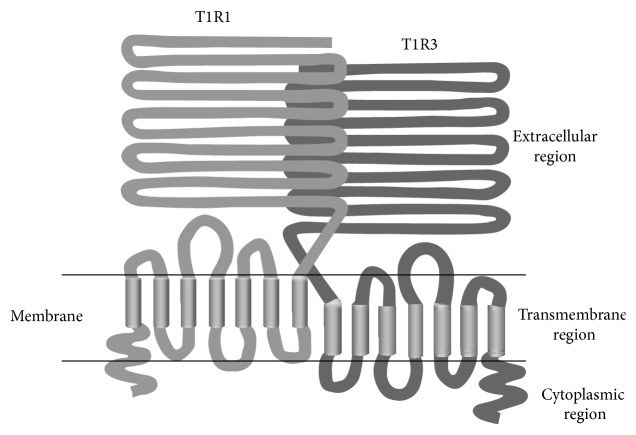
Schematic structure of T1R1 + T1R3.

Later T1R1 + T1R3 (Figure 8) was identified to be an amino acid receptor [29]. This receptor identified from mice showed synergism between 5′-inosinate and not only glutamate but also many other amino acids. This is consistent with the recording of taste nerve responses in rat [12] and mice [29], although in human, the synergism is seen between 5′-inosinate and only glutamate. Human T1R1 + T1R3 was produced and this receptor showed the synergism between 5′-inosinate and only glutamate [30]. The behavior of this receptor is consistent with psychophysical data in human and then T1R1 + T1R3 was established to be umami receptor in human.

T1Rs including T1R1 and T1R3 belong to family C of GPCRs and have three regions: the large extracellular region, the seven-spanning transmembrane region, and the cytoplasmic region [31]. The extracellular region is further divided into the ligand-binding region, which is frequently called the “Venus flytrap module,” and the cysteine-rich domain, which intervenes between the ligand-binding and transmembrane regions.

By site-directed mutagenesis and molecular modeling, binding sites for glutamate and 5′-inosinate in human umami receptor were clarified to exist in Venus flytrap of T1R1 subunit [32]. Here glutamate binds close to the Venus flytrap along the hinge-bending motion, which leads to stabilization of the active conformation. 5′-Inosinate binds to an adjacent site close to the binding site for glutamate. This leads to further stabilization of the active conformation. The structure of T1R1 is in dynamic equilibrium, where the ratio between the closed (active) and the open (inactive) conformations is modulated by the presence/absence of ligand [33, 34]. Thus the synergism is produced by an allosteric regulation.

### 8.3. Knockout Mice of T1R1 + T1R3 and mGluR4

Knockout mice of T1R1 and T1R3 were produced and responses to umami stimuli were examined by the measurements of nerve and behavioral responses. Knockout of T1R1 or T1R3 completely eliminated the response induced by synergism between glutamate and 5′-inosinate [35]. The response to glutamate alone was not examined.

Knockout mice of T1Rs were also carried out by other groups. In this case, knockout of T1R3 eliminated complete loss of the responses induced by the synergism [36]. But the response to glutamate alone was eliminated partly. That is, the knockout mice still responded to glutamate alone. Similarly knockout of T1R1 eliminated the responses induced by the synergism, but the response to glutamate alone was eliminated partly [37]. The remaining response to glutamate alone was reduced by addition of an antagonist of mGluR1 ((RS)-1-aminoindan-1,5-dicarboxylic acid) or that of mGluR4 ((RS)-*α*-cyclopropyl-4-phosphonophenylglycine). At the highest concentration of the antagonists, the response to 300 mM glutamate was reduced to 50–80% of control response. These results suggest that the receptors such as mGluR1 and mGluR4 also contribute to umami reception.

Knockout mice of mGluR4 were also produced and the responses to umami stimuli were recorded from taste nerves [38]. The knockout mice showed significantly smaller responses to glutamate than wild-type mice. The residual glutamate responses in the knockout mice were suppressed by gurmarin (a T1R3 blocker) and (RS)-1-aminoindan-1,5-dicarboxylic acid (an antagonist for mGluR1). These results provided functional evidences for the involvement in umami taste responses in mice. It is noted that as of today there is no report of the expression of mGluR1 and mGluR4 in human fungiform papillae.

The degree of the synergism between glutamate and the 5′-ribonucleotides greatly varies with species of animals. In dog, the synergism is much larger than that of rodents. As shown in Figure 6, addition of 5′-guanylate to a low glutamate of concentration which does not elicit the response induces a large umami response. Similarly, the synergism is very large in human as shown in Figure 2. In human, umami taste of glutamate alone is rather weak, but addition of 5′-inosinate increases umami taste severalfold. Umami taste induced by the synergism is essentially important in human.

It was reported that some humans cannot taste glutamate [39]. Kim et al. [40] examined variation in the human T1R taste receptor genes and showed that there was variation in genes T1R1, T1R2, and T1R3. Raliou et al. [41] found variation of genes T1R1 and T1R3 in human fungiform papillae and suggested that these receptor variants contribute to interindividual differences of sensitivity to glutamate. Thus T1R1 + T1R3 system mainly contributes to umami reception in human.

### 8.4. Transduction Mechanism

There are four types of taste cells [42]. Among them, type II and type III taste cells are able to transmit their signals to gustatory nerve fibers. Type III taste cells express synaptic vesicles, but type II cells do not possess conventional synapses but have very close contact with gustatory nerve fibers.

Umami reception is performed in type II and type III cells. Stimulation of umami receptor T1R1 + T1R3 by umami stimuli activates G-protein and leads to activation of phospholipase C*β*2 (PLC*β*2) [42, 43]. This activation produces inositol-1,4,5-triphosphate (IP_3_) that activates inositol-1,4,5-triphosphate receptor type 3 (IP_3_R3) to induce Ca^2+^ release from the Ca^2+^ stores. The increase in [Ca^2+^]i activates transient receptor potential of TRPM5 (transient receptor potential cation channel subfamily M member 5), leading to the depolarization of the taste cell. Finally, the taste cell evokes action potentials via voltage-gated Na^+^ channels and releases a transmitter to activate taste nerve fibers. The transmitter seems to be ATP [44]. It is also showed that glucagon-like peptide-1 (GLP-1) is secreted from taste buds by stimulation with umami stimuli [45].

Stimulation by umami stimuli of taste tissue brings about a decrease of cyclic AMP level [46]. Meaning of the cyclic AMP decrease is not elucidated.

## 9. Physiological Roles of Dietary Glutamate and Its Metabolic Disposition

There are glutamate receptors such as mGluR1 [47] and T1R1 + T1R3 [48] in stomach and intestinal epithelium. Stimulation of glutamate receptors by luminal glutamate activates vagal afferent nerve fibers whose information is transmitted to 3 areas of brains: the medial preoptic area, the hypothalamic dorsomedial nucleus, and habenular nucleus [49]. This stimulation seems to influence physiological functions such as thermoregulation and energy homeostasis.

The dietary glutamate including free glutamate and glutamate produced by digestion of food proteins is about 20 g per day [50]. The glutamate is adsorbed at small intestine. The adsorbed glutamate is extensively metabolized in first pass by the intestine. That is, most glutamate is used as a major oxidative fuel for the gut and metabolized into other nonessential amino acids. Glutamate is also an important precursor for bioactive molecules such as glutathione.

Since most glutamate adsorbed at small intestine is used as an oxidative fuel and metabolized into other amino acids, almost glutamate does not enter into the hepatic portal vein even when dietary glutamate is very high. Glutamate is a nonessential amino acid and then glutamate is synthesized in tissues such as muscle and brain. Glutamate is a neurotransmitter in brain and then brain contains glutamate in high content. Blood-brain barrier is impermeable to glutamate even at high concentration [51] and then dietary glutamate is not needed for brain.

In 1968 and 1969, two types of studies on safety of glutamate were reported. The first report was a very short letter on “Chinese restaurant syndrome” [52]. That is, eating of Chinese foods causes numbness at the neck and arms and palpitation, which is due to glutamate contained in the foods. Although no statistical data are presented in the letter, the news on the syndrome was spread all over the world. Later a number of double-blind placebo-controlled studies were conducted with the subjects who reported the syndrome and it was concluded that there was no relation between glutamate intake and the syndrome [53].

Content of the second report was that injection of a high concentration of glutamate into newborn mice induced neuronal necrosis in several regions of brain [54]. However, to evaluate safety of food components by injection is unreasonable because injection is quite different from oral administration. For example, injection of KCl contained in many foods such as an apple to animals leads to immediate death. As mentioned above, mother milk contains high concentration of glutamate and hence baby drinks a high concentration of glutamate every day.

In 1987, Joint FAO/WHO (Expert Committee on Food Additives) determined that acceptable daily intake of glutamate is not specified.

## 10. Discussion and Conclusion

The results obtained from rodents are not simply applicable to human because there are large differences between taste system of rodents and that of human. (1) In human and dog, sodium chloride largely enhances the responses to amino acids, sugars, and umami substances, while such enhancement is not seen in rodents (unpublished data). (2) In rodents, 5′-inosinate enhances the responses to various amino acids, while 5′-inosinate enhances the response to only glutamate in human and dog. (3) The synergism between glutamate and the 5′-nucleotides is rather weak in rodents but is extremely large in human and dog. (4) There is a report saying that, in mice, a mixture of glutamate and 5′-inosinate is perceived as having a sweet or at least sucrose-like taste [55]. Of course, it is unlikely in human.

As shown in the present paper, umami taste was shown to be independent of the four basic tastes by psychophysical and electrophysiological studies. The receptors specific for umami were identified. Umami substances are found universally in many foods. Based on these facts, umami was internationally recognized as the fifth basic taste.

Different from the 4 basic tastes, umami does not exhibit extensive taste even when the concentration of umami substances is largely increased. Umami substances are contained universally in various foods. Umami taste harmonizes with other tastes in foods and brings about mildness and deliciousness.

mGluR1, mGluR4, and T1R1 + T1R3 were identified to be receptors for umami. T1R1 + T1R3 exhibits a synergism between glutamate and 5′-inosinate or 5′-guanylate, but mGluR1 and mGluR4 do not exhibit the synergism. Similar to dog, human exhibits an extreme large synergism although glutamate and 5′-nucleotides alone exhibit only a small umami taste. Since glutamate and 5′-inosinate are contained in various foods, we taste umami induced by the synergism between glutamate and 5′-inosinate in daily eating. Hence T1R1 + T1R3 mainly contributes to umami taste in human.

A large amount of glutamate which comes from free glutamate in foods and digestion of proteins in foods is adsorbed at small intestine. Most glutamate adsorbed is used as major oxidative fuel for the gut, metabolized into other nonessential amino acids and production of glutathione. Almost glutamate does not enter into the hepatic portal vein even when dietary glutamate is very high. Safety of glutamate was confirmed by Joint FAO/WHO (Expert Committee on Food Additives).

## Figures and Tables

**Table 1:** Contents of umami substances in various foodstuffs [10].

Glutamate (mg/100 g)					
Plant		Animal		Traditional foods	
*Kombu *	1200–3400	Scallop	140	Anchovies	630–1440
*Nori* (seaweed)	1380	Kuruma shrimp	120	Cheese	300–1680
Tamarillo^*∗*^	470–1200	Sea urchin	100	Fish sauce	620–1380
Tomato	150–250	Short necked clam	90	Soy sauce	410–1260
Macambo^*∗∗*^	220	Crab	20–80	Green tea	220–670
Garlic	110	Egg yolk	50	Aged cured ham	340
Potato	30–100				
Chinese cabbage	40–90				
Carrot	40–80				
Onion	20–50				

		5′-Inosinate (mg/100 g)		5′-Guanylate (mg/100 g)	

		Dried bonito	470–800	Dried *shiitake *	150
		Dried sardine	350–800	Enoki (cooked)	50
		Yellowtail	410–470	Dried morel	40
		Sardine	420	Dried porcini	10
		Sea bream	180–400		
		Tuna	250–360		
		Chicken	230		
		Pork	230		
		Beef	80		

^*∗*^Relative to cacao plant.

^*∗∗*^Relative to tomato.

**Table 2:** Essential components for crab meat taste [11].

Component	Concentration (mg/100 mL)	Role of component
Glycine	600	Characterization of taste of crab meat
Alanine	200	
Arginine	600	

Glutamate	30	Addition of umami
5′-Inosinate	20	

NaCl	500	Enhancement of tastes of amino acids and umami substances

K_2_HPO_4_	400	
